# Penalties and Crimes.—A Symposium

**Published:** 1882-04

**Authors:** W. E. Knox, Thos. K. Beecher, Jas. B. Chandler


### PENALTIES AND CRIMES.

**Fines, Imprisonments, Hard Labor, Political Disability, Death.**

*Can this list be profitably enlarged? Shall physical pains be added to the list—corporal punishment—whipping, etc? Shall public exposure, as in pillory or stocks be used to cause shame? Is death the supreme or capital punishment?*

### A SYMPOSIUM.

The invitation to the symposium found me away from home, in circumstances not convenient to accept it, and with the shortest allowance of time for sending in the needed copy. Rather than not have the table furnished with guests, I accept the chair offered me, knowing that what I may crudely furnish will be supplemented by the more deliberate and matured utterances of the agreeable company with whom I am associated.

All law must have its penalty, else it is mere advice. The civil, moral and natural codes are alike in this respect. Their statutes may be disobeyed, but there stands the *caveat;* beware of the consequences! The penalty expresses the value of the law and the corresponding heinousness of the offence; it enforces future obedience on the offender, and makes his punishment a healthful monition to others not yet inculpated. It asserts also the existence and authority of the government and the respect due to its majesty. Are we not all agreed in these offices of penalty? That it is reformatory in its intent no one denies; that it is exemplary also in its influence, who can doubt? But where reformation is out of the question, the law cannot let go its hold. It only the more vigilantly guards and deeply incarcerates the man who is too thoroughly depraved to be reformed, and whose punishment will probably have little effect upon other natures disposed to the same crimes. Thus, though the penalty may have no redeeming or monitory effect as regards himself or others, it must still be enforced against him if the government have any regard to its own sacredness or to the expression of its righteous abhorrence of evil-doing.

It is a more difficult question what the proper penalties are for effecting the ends I have specified, and especially for the end had chiefly in view in this discussion—the *reforming* office of penalty. The usual penalties are readily enumerated, viz: imprisonment, hard labor, fines, political disfranchisement, death. I am asked by the symposium chair whether this list can be profitably enlarged; whether physical pains shall be added, such as corporal punishment, including the whipping post; whether public exposure, as in the pillory and stocks, should be used to cause shame, and whether death is the supreme or capital punishment. I have need to be a wise man to answer all these questions wisely. Briefly as I can, I will touch upon some of them.

*Can the list of penalties be profitably enlarged?* That depends very much on the answer to another question, whether the present list of penalties can be wisely enforced. That they are not is too evident, and hence the query whether others may not be brought in to supply their lack. We have imprisonment and hard work, but do we not need the pillory and the whipping post? I would say yes, if imprisonment and hard work have done their best in the way of penalty. For some criminals ten minutes at the whipping post would be worth more than ten days or thirty in most of our jails. It would leave a more memorable impression on the offender, and convey a sharper warning to the beholder.

For one office of imprisonment is the restraint of liberty, and to the average American citizen this should be a severe penalty. But the jail bird is not an average citizen, and what if to him imprisonment is a donation of liberty instead of a deprivation of it! Liberty, means of living at the public expense, and not by his own toil; liberty of shelter from cold, of relief from hunger; liberty of genial companionship with a herd as depraved as himself. What is there in this of the nature of penalty? It is a well known fact that some of our vagrant population commit crime as December comes on that they may have the benefit of better winter quarters than they can find at home. If any one doubts that we have such persons in Elmira, let them apply to the City Missionary for the names, and get their doubts resolved. What if it were a naked whipping post such worthies were introduced to instead of well warmed apartments in our city penitentiary. One class of crimes would disappear on the first trial of the new remedy.

Again: Imprisonment should work a salutary sense of shame in the breast of the culprit. But what if it helps to obliterate all such feeling? The young criminal, mortified at his first detection, has a hang-dog look as he takes his place behind the prison grates. Imagine his surprise to find his entrance greeted with cheers, his misgivings and misery treated as a good joke. He cannot most likely take that view of the case at once, but thirty days' schooling with these associates will aid wonderfully in acquiring their lesson and teaching it to new-comers.

Still further: Imprisonment is intended as a protection to society by removing unworthy members from the contact with the body politic and the consequent contagion of their presence But how little this end is secured if imprisonment, by herding the old and young offenders together, affords the best opportunity Satan could ask for of rendering those young in sin old before their time, and by the shortest process possible.

Another most important point: Idleness is, of all causes of crime, perhaps the most fruitful. The criminal classes do not love work. Regular ocupation by the day and month and year is a terror to them. Imprisonment, therefore, should do another office in putting them in a way at once to earn their own living and refund the State the cost of their arrest and conviction. But this it plainly does not do in the multitude of instances where no work is required of the criminal; but, on the contrary, a free support is accorded him at the public expense. Better a hundred times the pillory than this promiscuous herding of men in idleness, since solitary posing for hours and days in the Simon Stilites fashion would be a labor to these fellows beyond what it was to devout Simon, and accompanied, we may hope, with some sense of shame such as that very model saint would not have regarded it consistent to feel.

So much for the question whether our present penalties are wisely enforced. I have confined the question to imprisonment and labor, because the former is the most common form penalties takes, and the others, if you except the death penalty, are subordinate. Few criminals, I imagine have found any deterrant motive in the prospective political disability the law threatens, so much more prominently do other possible losses enter into their estimate. Fines have no doubt a stronger consideration with some, yet the number is comparatively few to whom that penalty applies, and in their case it is often mitigated by the hope of a pecuniary profit from their criminal act, more than sufficient to reimburse the governmental exaction.

I have also had jails and not prisons chiefly in my eye thus far, because the illustration from that question is so conspicuous, and the defect of our criminal policy so vital. The jails altogether contain the larger proportion of offenders, as is evident from the number of the jails, and the short stay of the tenants who come and go in rapid succession, such as does not pertain to the more populous penitentiary. The jail is the feeder of the prison. It not only holds in temporary keeping, those who are awaiting trial for graver crimes, but a multitude committed for petty offences who are in training there for felonies that shall hereafter graduate them into state prison. If prison reform is needed, how much more jail reform, whether the area of penalty should be enlarged or contracted, cannot be determined without taking into permanent account the operation of punitive agencies within the walls of the city lock-up and county jail.

As to our State penitentiaries the outlook toward reform is hopeful, and suggests no need of new penalties. The convicts are not allowed in company, except for work, worship or meals, unless where, as in our Reformatory in Elmira, they are associated for study. The rule is also that each one shall be supplied with work. Where is the need here for pillory or whipping post? The need is in another direction; the intermixture of new rewards with the old penalties. We are attaining to a condition in which our convicts are put upon the means of self support, we need to associate with it another in which they shall be placed under motives to the best behavior. Such a system has been admirably perfected, and set in operation in the Elmira Reformatory, by the establishing of grades or sections, that indicate the moral standing of the convict, and the abridgement or lengthening of his period of confinement. Mental and moral instruction is furnished, and a supervision that is not merely perfunctory, but personal and parental, If the clamor of demagogues can be kept down, and the good sense of our best citizens maintained in the ascendancy, we may expect great results from the new departure our State has taken in the matter of prison reform.

My answer to the first question has included in it the reply to the second and third. "*Shall physical pains be added to the list?*" "*Shall public exposures as in pillory, stocks, &c., be used to cause shame.*" I say *no*, if the penalties already on the list be wisely used. Imprisonment can be so managed as to produce shame, so far as needed in a large number of convicts. Where there is a failure let the form be varied. It would be an edifying spectacle if the idle crew in most of our city jails could be set at work in chain gangs on the streets. A few days of that kind of discipline would thin out the commitments more than six months incarceration with labor. Plenty of hard work would quickly rid the public of that class of beneficiaries who go to jail for the sake of bed and board. "Something for nothing," is the rule by which our vagrant population seek their living. This is at the root of all our beggary. This is the main opening of most of our crime. This is why so much of our taxes are wasted by commissioners of the poor on many persons who have no more claim on the donation then the commissioners themselves. Look at the thousands of dollars yearly spent in this city, on able-bodied paupers, who increase their demand in the ratio of supplies, and who demand mendicant relief in return for political support. Do not their votes make the commissioner and shall "the thing formed say to those who formed it, why do ye thus?"

Touching the last question *whether death is the supreme or capital punishment*. I have room but for a word.

I answer yes, if it most adequately expresses the value of life and the heinousness of the crime that destroys life. There is no safeguard too strong to set about the life of the citizen. The motive which will best hold back the murderer's hand from his deed, is the one that should be brought to bear upon him. Is there any other that can compete in this respect with the death penalty? I believe not. If there is any other, it is imprisonment for life. In the judgment of the country in general, or of the criminal himself can there be any doubt that imprisonment for life is no substitute for punishment by death, above all that it is not a severer substitute?

If life imprisonment is the severer, why is it that criminals always prefer it to the milder one—why are they always exhilarated with the hope that the Governor may commute the hanging just at hand for the life sentence to Auburn? Why do their lawyers never ask the jury to bring in a verdict for the gallows rather than the prison? why do they never prefer the indictment of man-slaughter? why is the community never heard condemning the sentence to imprisonment for life as too severe, and insisting that the milder one of capital punishment should have been pronounced in its place? These questions answer themselves. It is only when men have a theory to defend that they argue for life-imprisonment as the milder punishment, when it comes to a practical instance their instincts and acts are all the other way.

W. E. KNOX.

---

Thus far in time and history men *under compulsion* have attained to higher strength, intelligence, and virtue than men at ease, and *free to choose.* Switzerland, Scotland, New England and other such regions used to say "*work for a living or take the consequences.*" This law of work enforced by frost and hunger, impartially administered upon all has proved to be salutary. And in general, I note as to all the uniform operations of nature—often called "laws," that men conform to them after a few experiments of insubordination.

Most boys burn off their eye-winkers once playing with powder; a few are so heady as to burn themselves twice; but generally, they learn wisdom by the things that they suffer. The *habit* of tickling a mule's hind legs with a straw, rarely enslaves the moderate tickler, however hilarious. Thus men are educated under law—the inexorable law of cause and effect, conduct and consequences. And so long as men are dealing with matter, and force—NATURE—they are inexorably and mercilessly governed. And thus far this school of compulsions by rewards and penalties, has yielded the very best results in the education of the faculties of man, considered as an individual.

By and by begins the association of individuals. Men draw together for the protection of life, property and the family. Society comes into being; usages come to pass and harden, and men begin to enact laws. The individual is no longer in account with NATURE only, but with his fellow man as well. The laws of NATURE (force and matter) need no executive officers. But the law of a society, large or small, stands on the will of a man, or men. Law is but a "King Log." The frogs hop over it, or squat on it, very much as Sunday papers treat Sunday laws, or bankers the usury laws, or politicians the anti-bribery laws, or drink-sellers the liquor laws. A law without executive energy is a watch-dog without teeth—a loaded rifle without trigger or marksman.

Here *again* is inserted the will of man. The law itself stood on the will of its enactors. The execution of it depends upon the will of the officers. Properly speaking that is not and cannot be an inexorable and impartial *law*, that owes its enactment and enforcement to the will of man. At best it is only consent or agreement. "Self-government" is no government. "Self-government" will, upon inspection, resolve itself into mere agreements—compromises. We must shack along together some how. The army, the navy, the railways, and other great organizations, are governments. Orders are issued, obedience is exacted. Grumble as you please, good sir, but obey or you'll catch it. Not so with society at large, plausibly described as a government of the people, by the people, for the people.

To what extent, then, it is practicable to imitate the salutary school of Nature, in the enactment and execution of Laws for society is a question difficult to answer.

Were it practicable to fix things so that he who shoots or stabs shall drop dead in his tracks, all murders would cease from off the earth at once. But human law thus ordaining, is met by cushions of delay and thwart: 1. Can you catch him? 2. Can you prove the offence? 3. Is your judge stern and dispassionate? 4. Is your jury impartial and loyal to duty? 5. Is the prisoner of sound mind? 6. What says public sentiment?

So again, of physical pain: NATURE burns, gashes, bruises; and by aches, twinges, cramps and anguish works repentances and reforms. The cruelty of man has never equaled the ingenuity of NATURE in the variety and intensity of torment inflicted upon law-breakers.

Can human law profitably borrow a leaf from the law-book of Nature? Shall we bring back corporal punishment? of course we answer—Yes! provided we can imitate the inexorable, cause-and-effect quality of NATURE'S laws. But here again come in our cushions of delay and damage. When a teacher flogs a boy, or a warden spanks or paddles a prisoner, the pain inflicted is far less than an ordinary attack of colic, the penalty of green-apples stolen! But it seems to be gratuitous, cruel, and all that. The flogging stands in the will of man. Let's argue the matter in the newspapers!

The point I urge is that corporal punishment is salutary when inflicted by NATURE. And from a scientific point of view, *we* are logically bound to learn of her—the only true goddess—and make our laws conform to the laws of Nature, death penalty included. What considerations I should urge were I writing as a Christian teacher true to my convictions, is another question—not here answered.

I conclude, that if laws can be inexorably administered, they will work good results on human nature. Also that pain and death are indicated as among the most salutary of penalties; and that they should be freely imitated, transferring them from the ordinances of NATURE, and placing them among the statutes of men.

But if such laws cannot be mercilessly executed, because of certain hindrances lodged in human nature, then open the vast and higher ranges of influence and education among men, long ago suggested in the new commandment, that ye love one another.

THOS. K. BEECHER.

---

Laws against crime are founded upon the necessities of communities, and, although varying with localities, they are all intended for the preservation of peace and harmony and the punishment and reformation of evil doers.

If a penalty can be found which will prevent a crime, that penalty should be attached to the law which makes a crime; for, be it remembered, a new law makes a new crime, and, as legislators are constantly at work, the chances for law breaking become more numerous.

*Fines.*—This form of punishment has strong advocacy in the fact that so many individuals consider the real *argumentum ad hominem* to be that which assails the pocket. Alone, it can only be justly and safely applied to a few minor offences against municipal ordinances, and even then it is but a nominal punishment to the rich, whilst it may be a serious matter to the poor, whose temptation to commit the prohibited act may be equal, if not greater.

When the privilege to commit a crime may be purchased by paying a fixed fine, then the gratification of the wish becomes merely a question of economy. Instances are on record of the evasion of ordinances by the prepaying of the fine demanded for the overt act.

*Imprisonment.*—This branch of the subject needs volumes instead of pages for its proper discussion. The mere question of whether or not a criminal should be incarcerated requires no argument. The real question is, whether the imprisonment is intended to be merely the tying up of the dog to keep him from biting others, the locking up of a thief where he cannot steal, or whether only as a deprivation of liberty, a mere punishment for an offence; or, still further, as a means of reformation of the prisoner and a warning to others intending evil.

As I understand it, the scope of this article is merely the discussion of the values of existing legal penalties and the suggestion of others.

To many, imprisonment would be a terrible punishment; but, unfortunately, to a large number of the crime-class life in prison is luxurious when compared with their existence in the slums of large cities when not "doing time."

The prison records of New York and Philadelphia contain the names of thousands of men and women who are almost constant inmates of cells. They serve out their "time" on one sentence, and in a week or two return to prison by a repetition of the old, or the commission of a new, violation of law. They are literally "jail birds," and flock *home* after their holiday and debauch to recover strength and health from regular diet, clean beds, comfortable quarters and good medical attendance.

Compare these advantages of the prison with the discomforts of the dens and cellars of the purlieus and haunts of vice in a large city, and is it any wonder that the prisons are full and the price of admission is paid by the breaking of the laws?

At the cell doors of the Philadelphia county prison slates are hung, upon which are written the name of the inmate and the number of times committed. I have seen that number as high as thirty-two! In that particular case it was a woman. Rum and fighting were her passports to the cell. I saw her released one morning after the matron had provided her with her out-of-prison dress. She was a comparatively young woman, good looking, bright, civil spoken, and went from the prison to take a place as servant, which her sister had procured for her. She bade all good-bye, and said she had firmly resolved never to return.

That afternoon she was "brought in" beastly drunk; had managed to get into a fight and back to prison all in the space of a few hours. A douche brought her to reason and decency; she was clad in her prison dress, and returned to the cell. "Thirty three" was substituted for "thirty-two" on the slate, and I registered another claim for corporal punishment.

Imprisonment, whether its purpose be mere punishment, prospective reformation, or to secure honest people from the depredations of the criminal whilst incarcerated, should be under a carefully digested system of some kind, and that system ought to be carried out faithfully by officers and servants of special training for the work. Otherwise, favoritism, political preference and other bad influences may creep in to prevent justice; and, on the other hand, starvation and cruelty may serve to gratify revenge for fancied or real injuries or insults to those in power.

The association of prisoners, the old professional cut-throat and thief, with the younger beginner in the walks of crime, is to be deprecated as a school for vice, from which the pupil graduates, at the conclusion of his short term, educated to make his mark in higher criminal ranks.

As an advocate of capital punishment I have but little sympathy with life imprisonment, a crime demanding that demands more.

*Hard Labor.* What is known as hard labor in our prisons is usually nothing more than employment, and to a prisoner of heretofore active habit the want of it would be additional punishment.

There are instances now, and I think they might be profitably increased, where prisoners perform the heavy manual labor required in public works, cleaning streets, sewers, &c., or the labor on farms, in quarries or mines, breaking and laying stone and other outdoor hard work. These employments do not bring honest tradesmen in competition with convict labor, and I can see no strength in the argument that the convicts are thereby exposed to public gaze, and shame added to their punishment.

*Political Disability.* This penalty should accompany more sentences than it now does. The man who will deliberately break an important law, is certainly not fit to have any say in the making of laws, and therefore, for the general good, should be deprived of voting and from holding official station. Especially should this penalty be applicable to all election frauds.

*Death*—Or as it is generally known "Capital Punishment." The list of crimes for which this penalty is awarded has been most unadvisedly very much reduced of late years. The consequences are manifested in a large proportionate increase of those crimes, such as highway robbery, arson, rape, and certain, so called, degrees of murder—nevertheless murders have not decreased, and therefore tender hearted people whose sympathies have been awakened by the seeming repentance of the condemned, or the piteous mourning of his relatives, argue that the death penalty should be abolished. This sympathy is misplaced, the finding out and condemnation are the repented points, not the death of the poor victim. After, under a just judge with a fair trial, the accused has had opportunity to show all extenuating causes if there have been any, which led to the murder, and in spite of these his guilt has been established and sentence passed, then public weal demands the execution, not entirely upon the *lex talionis*, but to rid the community of a murderer and to warn others from the crime.

1. *Can this list of penalties be profitably enlarged.* It seems as if there were wanting some other penalties to meet minor offenses, which now either go unpunished, or imprisonment is inflicted to the great injury or disadvantage of innocent parties. A woman taken from a family of young children for instance ; she may deserve punishment and require it for the future comfort of her neighbors, but her presence at home is absolutely necessary to the family and they will suffer. A profitable enlargement of the list might be made to meet such cases. Whether it should be in the shape of the obsolete "Ducking Stool," or some other quick personal chastisement is a problem for solving. Places of correction, not exactly prisons, yet having authority of detention are being tried in some States with fair results, and also with occasional disrepute from questionable management.

There is a question in this connection which might be discussed with possible benefit, had we space; that is, whether or not the detected and defeated planner of a crime should not be punished to the extent of the law as if his plans had been successful. The attempt to wreck a passenger train for plunder is an example of such a crime.

2. *Shall physical pain be added to the list ; corporal punishment, whipping, &c.* This question has been fully answered in the State of Delaware. In spite of morbid, miscalled philanthropic movements from other quarters against it, backed up by attacks from the press, the people of Delaware persist in a punishment which greatly reduces their crime rate.

The problem of relieving prisons of their *constant* population has been seriously labored with by those who from frequent visitation, are familiar with the fact, that they are made free boarding houses for lazy, vicious, vagabonds, who will not work except under coercion, and who, when out of jail will commit any crime, which will yield the means for debauch until caught, and which carries with it no greater danger than a return to their old comfortable prison quarters for a specified time. These people have been dealt with by reason, kindness and good advice from persons capable from a lifetime spent in the labor, yet the harvest brings so little good result, that even the faithful philanthropists are becoming advocates for some corporal punishment, which will appeal to animal senses, all others seeming lost, (if they ever existed) in a life of debauchery and crime.

"Did you ever get caught in Delaware," I asked of a frequenter of the Dock. "Yes, once," he answered, "but it was a mistake, I thought I was operating over the State line, in Pennsylvania. *I have reformed in Delaware.*"

The dread of the whipping-post will deter many from crime whose only hesitancy is the fear of detection and dread of corporal punishment. Fines will not reach them, for they have nothing to lose. Imprisonment has no horrors, except the lack of excitement and the temporary restraint from the gratification of bad instincts. If our prisons are, on the claim of humanity, to be conducted on a scale of comfort and convenience exceeding in many instances the hotels which travelers too often encounter, some other punishment must be found for the idle and vicious, if idleness and viciousness are to be cured.

3. *Shall public exposure, as pillory or stocks, &c., be used to cause shame?* I doubt the propriety of the revival of the pillory and stocks, but there can be no reason why, in the case of confirmed and uncontrollable criminals, the ball and chain should not be used, if necessary, when they are employed on public work outside the prison, and I think experience in older countries warrants the chain gang system here.

4. *Is death the supreme or capital punishment?* The question of which is the highest or severest punishment must depend upon the punished. The circumstances which have surrounded him; the character and effect of early teachings; the settled or unsettled convictions he may have as to a future state of reward and punishment; or as to the efficacy of atonement, or intercession and other matters of religion, as well as secular and worldly ties. The Japanese, who for a sum of money, in many instances trifling, paid to his family, will offer himself a substitute for a richer man condemned, would not do so if the sentence were a severe flogging instead of death. The religious enthusiast, who would murder a heretic to his particular creed, would go up to the scaffold glorying in the deed and his martyrdom. The man whose life has been one of hard toil in the mine, and who has never known pleasanter emotion than bad whiskey gave, who under a voluntary submission to his Order could deliberately shoot down a fellow workman supporting his little family at the highest price obtainable for his labor, will, after sentence and the consolations from the religion of his childhood, walk proudly to the scaffold and assure his neighbors and friends that he has made his peace with God, and swing off into eternity the hero of the hour. Contrast such a death-scene with that of his victim, perhaps one whose religious convictions were the same as those of his murderer, but whose speedy taking-off gave only time to realize that he was dying without those, to him, very essentials for heaven; "unhousel'd, disappointed, unanel'd."

Perhaps just here might come an argument for the restoration of the old penalty, "without benefit of clergy." It would be a severe sentence for some, but, like the death penalty, would have little terror for many others. May not the chances for confession and absolution between sentence and execution be factors in some deliberate plans for murder?

The severity or quality of any punishment, I repeat, depends so largely upon the character of the convict, that what would be "Supreme" to one, would be the preference, or rather choice in the list, of another. Death to many would be more easily endured than lengthened imprisonment or even the lash.

Before closing this, I would ask special attention to the necessity for enlarged discretion on the part of the judge--what would crush one man, would be but a bagatelle to another, therefore, the laws should be so framed as to admit of gradation of punishment, and the judges should be carefully selected, not only with a view to legal talent and probity, but especially from those who possess sound discrimination and good judgment of men.

JAS. B. CHANDLER.

